# *Notes from the Field:* Enteroinvasive *Escherichia coli* Outbreak Associated with a Potluck Party — North Carolina, June–July 2018

**DOI:** 10.15585/mmwr.mm6807a5

**Published:** 2019-02-22

**Authors:** Carolyn T.A. Herzig, Aaron T. Fleischauer, Brian Lackey, Nicole Lee, Thomas Lawson, Zack S. Moore, John Hergert, Victoria Mobley, Jennifer MacFarquhar, Tammra Morrison, Nancy Strockbine, Haley Martin

**Affiliations:** ^1^Epidemic Intelligence Service, CDC; ^2^Division of Public Health, North Carolina Department of Health and Human Services, Raleigh, North Carolina; ^3^Center for Preparedness and Response, CDC; ^4^Mecklenburg County Public Health, Charlotte, North Carolina; ^5^Division of Foodborne, Waterborne, and Environmental Diseases, National Center for Emerging Zoonotic and Infectious Diseases, CDC.

On July 2, 2018, the North Carolina Division of Public Health was notified that approximately three dozen members of an ethnic Nepali refugee community had been transported to area hospitals for severe gastrointestinal illness after attending a potluck party on June 30. The North Carolina Division of Public Health partnered with the local health department and CDC to investigate the outbreak, identify the cause, and prevent further transmission. The investigation included molecular-guided laboratory testing of clinical specimens by CDC, which determined that this was the first confirmed U.S. outbreak of enteroinvasive *Escherichia coli* (EIEC) in 47 years.

A case was defined as the occurrence of diarrhea, vomiting, or fever ≥101°F (38.3°C) in a person who consumed food served at the party. Cases were identified through medical record review and retrospective cohort investigation with convenience sampling of party attendees. Among approximately 100 attendees, 52 met the case definition. Median age was 31 years (range = 3–76 years); 28 (54%) were hospitalized, including 13 (25%) with sepsis, and eight (15%) who were admitted to an intensive care unit. All patients recovered, and no secondary cases were identified.

Forty-nine persons, including 35 who were ill, were interviewed using a questionnaire to ascertain symptoms, recent travel, and food exposures. Participants also were provided with hand hygiene guidance. Among the 35 ill persons, 30 (86%) reported symptom onset on July 1, the day after the event (Figure). Median interval between eating and symptom onset was 20.5 hours (range = 1–45.5 hours). Overall, 33 (94%) ill persons experienced diarrhea, including 27 (77%), 19 (54%), and two (6%) who reported diarrhea that was watery, mucoid, or bloody, respectively. Thirty-two (91%) ill persons reported fever.

**FIGURE:**
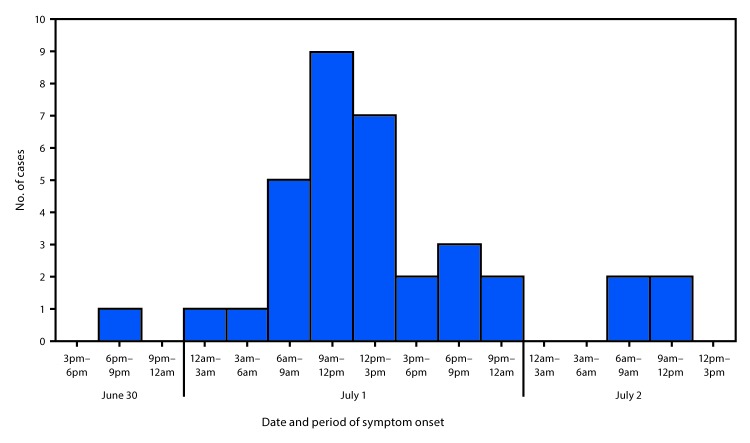
Number of enteroinvasive *Escherichia coli* cases (N = 35), by reported date and period of symptom onset — North Carolina, June 30–July 2, 2018

Participants reported eating chicken curry, vegetable curry, rice, lentil soup, fried bread, cold and hot salads, and cake; no imported foods were reported. No single food item was statistically significantly associated with illness; however, 37 persons reported eating chicken curry, and those who did had a 47% higher risk for illness than those who did not (risk ratio = 1.47; 95% confidence interval = 0.76–2.83). No food was available for testing. No interviewed person reported recent travel.

One hospital used a commercial multiplex polymerase chain reaction (PCR) gastrointestinal panel to test stool specimens from 25 patients; all tested positive for *Shigella*/EIEC (*Shigella* and EIEC are difficult to differentiate in clinical specimens, and the commercial panel does not distinguish between the two). Twenty-four of these specimens were submitted to the North Carolina State Laboratory of Public Health, and all were negative for *Shigella* and *E. coli* O157 by culture and for Shiga toxin genes *stx*_1_ and *stx*_2_ by PCR. MacConkey broths and stool specimens from 23 patients tested at the state laboratory were submitted to CDC, where a molecular-guided approach was used for the isolation of *Shigella*/EIEC. Colonies positive for *ipaH*, the target gene for *Shigella*/EIEC, were identified as EIEC O8:H19 in specimens from 12 patients.

This was the first confirmed outbreak of EIEC in the United States in 47 years, and the first report of EIEC serotype O8:H19. EIEC is a human enteric pathogen that causes dysentery and is transmitted through contaminated food or water and person-to-person contact (*1*). Infections occur most commonly in developing countries (*1*,*2*). The last known outbreak in the United States was reported in 1971 and was associated with imported cheese (*3*). More recently, contaminated vegetables were implicated in outbreaks in Italy in 2012 and the United Kingdom in 2014 (*4*,*5*). This investigation did not reveal the specific vehicle through which EIEC was transmitted.

*Shigella* was initially suspected based on preliminary PCR results and because EIEC infections are rarely identified. However, epidemiologic and clinical findings were inconsistent with previous *Shigella* outbreaks in North Carolina, which are typically associated with person-to-person transmission in child care settings and less severe clinical manifestations. Because of genetic and pathogenic similarities, EIEC and *Shigella* can be difficult to distinguish (*1*), and identification required a molecular-guided approach. This investigation highlights the importance of collaboration between epidemiologists and laboratorians when findings are inconsistent with the initial suspected etiology.
